# Continuous Professional Competence (CPC) for Irish paramedics and advanced paramedics: a national study

**DOI:** 10.1186/1472-6920-14-41

**Published:** 2014-03-02

**Authors:** Shane Knox, Walter Cullen, Colum Dunne

**Affiliations:** 1 Graduate Entry Medical School and Centre for Interventions in Infection, Inflammation & Immunity (4i) University of Limerick, Limerick, Ireland; 2 Health Services Executive, National Ambulance Service College, Dublin, Ireland

**Keywords:** Paramedics, Advanced paramedics, Continuous professional development, CPD, Continuous professional competence

## Abstract

**Background:**

Internationally, continuing professional competence (CPC) is an increasingly important issue for all health professionals. With the imminent introduction of a CPC framework for paramedics and advanced paramedics (APs) in Ireland, this paper aims to identify factors that will inform the implementation of this CPC framework by seeking stakeholder input into the development of a CPC model for use by the regulatory body. Our secondary objective is to determine the attitudes of registrants towards CPC and what they consider as optimal educational outcomes and activities, for the purposes of CPC.

**Methods:**

All paramedics and APs registered in Ireland (n = 1816) were invited by email to complete an anonymous on-line survey. The study instrument was designed based on CPD questionnaires used by other healthcare professions. Quantitative and qualitative analyses were performed.

**Results:**

The overall response rate was 43% (n = 789), with 82% of APs and 38% of paramedics participating. Eighty-nine per cent agreed that registration was of personal importance; 74% agreed that evidence of CPC should be maintained and 39% believed that persistent failure to meet CPC requirements should mandate denial of registration. From a pre-determined list of activities, respondents indicated practical training scenarios (94%), cardiac re-certification (92%), e-learning supplemented by related practice (90%) and training with simulation manikins (88%) were most relevant, while e-learning alone (36%), project work (27%) and reading journal articles (24%) were least relevant.

**Conclusions:**

Irish Paramedics and APs are supportive of CPC linked with their professional development and registration. Blended learning, involving evidence of patient contact, team-based learning and practical skills are preferred CPC activities.

## Background

In 1993, a report from the Irish Government [1] recommended a significant improvement in the quality of training provided to ambulance personnel. This recommendation was reiterated most recently in the PHECC strategic plan (2011-2014) where the need to develop and implement a continuous professional competence (CPC) framework was stated [2]. This task is made more difficult in Ireland as, currently, once qualified there is no regulatory requirement for the practitioner to provide evidence of competence, or any link between competence and registration to practice. However, it is reasonable that practitioners and consumers alike view maintenance of competency as a basic element of ethical and responsible practice [3].

One of the functions of a healthcare Regulator is to protect the public by ensuring that acceptable standards of care are being provided [4]. Previous studies have assessed emergency medical technician, paramedic and advanced paramedic (AP) training and continuing education in Ireland [5,6] and internationally [7-9]. However, in this study we wished to determine, for the first time, the attitudes of Irish paramedics and APs towards CPC, their preferred activities, delivery format and relevance to their roles.

It has been stated that any form of compulsory education is incongruent with the nature of both being a professional and adult; professionals should be self-directed enough to participate autonomously in educational activities rather than being compelled [10].For Continuing Medical Education (CME) to be effective, Norman *et al*. believe that there is a requirement to justify CME content through a specific needs assessment [11]. With this in mind, we aimed to devise a short answer survey to guide and inform the impending CPC implementation in Ireland.

## Methods

### Context

The majority of front-line emergency ambulance services in Ireland are provided by paramedics and supported by advanced paramedics. Advanced paramedics, having completed additional training, provide advanced life support skills and interventions while paramedics do not do so. Pre-hospital care in Ireland is largely provided by the Health Service Executive (HSE) National Ambulance Service (NAS) and (in parts of Dublin city) the ‘Dublin Fire Brigade’. Other providers of pre-hospital care include the Irish Permanent Defence Forces, Coastguard, and private ambulance services, in addition to Emergency Medical Technicians, mostly within the voluntary organisations: Civil Defence, Order of Malta Ireland, St. John Ambulance and the Irish Red Cross. All of these practitioners are registered with the regulating authority, Ireland’s Pre-Hospital Emergency Care Council (PHECC).

### Participants

In February 2012, all paramedics and APs licensed to practice in Ireland and registered with the Pre-Hospital Emergency Care Council’s (PHECC) with valid email addresses (n = 1816) were contacted and provided a link to the Survey Monkey™ online study instrument and to a concise, unbiased explanation of the survey topic. Participation was voluntary and anonymous. Consent to participate was recorded. As a follow-up, reminder emails which have been shown to be beneficial in improving the response rate [12] were emailed two weeks later to the same group. The design and conducting of the study, taking into consideration published healthcare professions’ questionnaires relating to continuous professional development (CPD) [13,14] were approved by the Ethics Committee of the Faculty of Education and Health Sciences, University of Limerick, Ireland and the Research Ethics Committee of the Health Services Executive Mid-Western Regional Hospital, Limerick, Ireland.

### Data collection and analysis

Health professionals are increasingly expected to identify their own learning needs through self-assessment [15]. Therefore, the survey questions were designed to elicit participants’ views on CPC. The survey was piloted by 20 registered paramedics who were subsequently excluded from the analyses.

The questionnaire was based on questionnaires used by other professions [13,14] comprised questions relating to: demographics; opinions regarding CPC, including the role of the employer; CPC portfolio development; linkage or CPC and registration. The response data were downloaded from Survey Monkey™ software to an electronic data file and quantitative analysis was performed using Statistical Packages for the Social Sciences (SPSS version 20.0). To make analysis more meaningful, responses to the five-point Likert scale were analysed using three options, ‘strongly agree/agree’, ‘undecided’ and ‘strongly disagree/disagree’.

## Results

### Demographics

**Figure 1:**
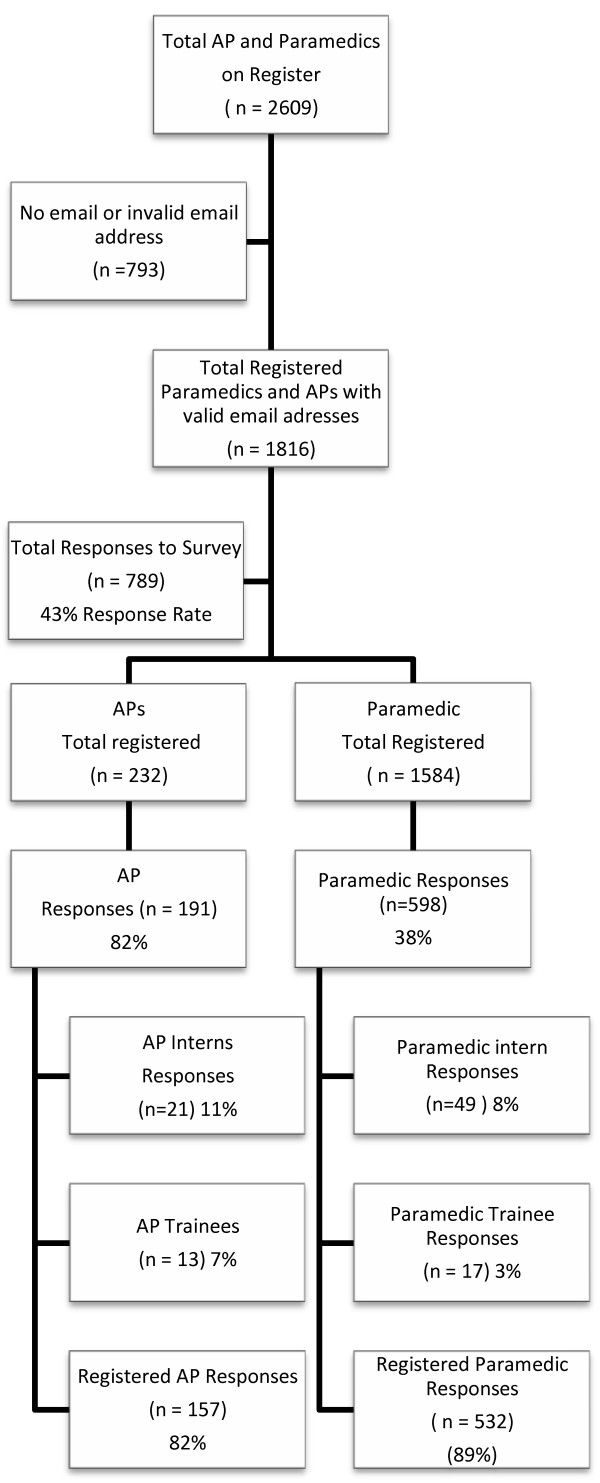
Registered paramedics and APs in Ireland and number of responses.

**Figure 2:**
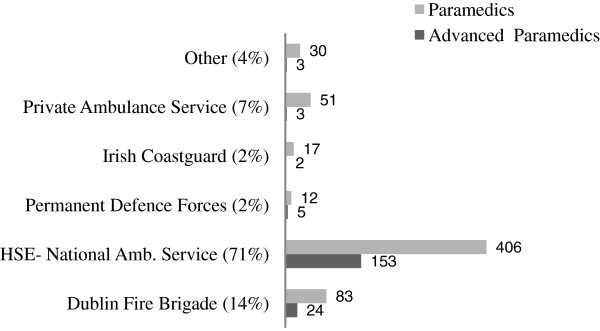
Responses from paramedics and advanced paramedics based on organisation (789 respondents).

789/1816 responses were received (43% of all registered paramedics and APs with email addresses), of whom 598 were paramedics. While the largest number of responses to the survey was from paramedics (38%, 598), 82% of the advanced paramedic cohort participated (191 of 232 APs) representing the greatest proportional response (Figure 1). The majority of respondents were male (85%, 670) (Table 1). These respondents predominantly served in the Irish National Ambulance Service (71%) and the Dublin Fire Brigade (14%) (Figure 2).

**Table 1:** Gender and registration level

**Registration status with regulatory body (PHECC)**	**Male**	**Female**	**Total**
Advanced paramedic	138	19	157
Advanced paramedic intern	19	2	21
Advanced paramedic trainee	11	2	13
Paramedic	451	81	532
Paramedic intern	38	11	49
Paramedic trainee	14	3	17
Response percentage	86%	14%	
Response totals	**670**	**119**	**789**

A total of 363 (46%) participants had been registered in Ireland for greater than six years, while 336 (30%) had been registered for 3-6 years and the remainder for less than 3 years. Respondents who had been with their organisation for less than five years represented 33% (260) of the total surveyed, while 15% (113) had over 20 years experience with their respective organisations.

### Attitudes towards continuous professional competence and registration

Registration with PHECC was considered personally important by 89% (697) of respondents, with only 2% disagreeing. Indeed, in the context of active professional pre-hospital practitioners, 77% (615) of the paramedics/APs stated that CPC was extremely important. Most respondents (74%, 584) agreed that CPC should be a condition of registration to practice, and 67% (526) agreed that paramedics and APs should maintain evidence of CPC activities to ensure registration, while only 8% (66) disagreed.

Further, 61% (487) of respondents believed that those practitioners who do not maintain CPC and continue not to meet the necessary requirements should not be allowed to re-register at their current level. 39% (307) agreed with the suggestion that those who fail to meet the CPC requirements should be allowed to register at the level below their current registration, but 23% (179) did not support this proposition. Of interest in the context of introducing a CPC framework for the first time, the majority of respondents (70%, 551) would not consider registering at a lower level rather than having to complete CPC.

### CPC activities

**Figure 3:**
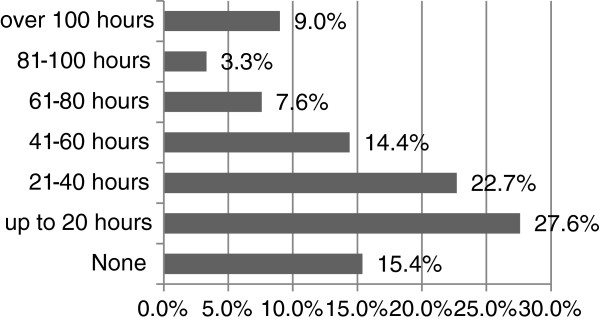
Number of CPC hours recorded in the previous 12-month period by APs and paramedics.

**Figure 4:**
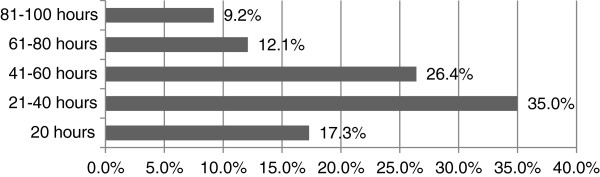
Number of annual CPC hours deemed appropriate by APs and paramedics.

A small majority of paramedics/APs surveyed (53%), although not obligated, maintained a professional portfolio at the time of the survey. Fifty seven per cent had completed greater than 20 hours of CPC activities in the prior 12 months, with nearly 20% (19.9%) having completed more than 60 hours (Figure 3). When queried as to appropriate levels of CPC required in a 12-month period, 35% believed 21-40 hours, 26% that 41-60 hours, and 17% that 20 hours would be adequate (Figure 4).

Forty per cent of those who had completed their CPC in the previous year had funded participation themselves, 23% had their costs covered in full by their employer, 9% had their costs partially covered and 12% had completed only cost-free CPC activities.

Sixty five per cent preferred funding to be provided for their own personal CPC activities so that they may decide what is relevant to their own needs, while 69% believed that the regulator should provide funding for organisations to develop their own customised CPC activities.

53% (422) of participants held the view that any individual’s CPC should be subject to audit by their respective organisations, while 38% (302) disagreed with the concept of CPC being the sole responsibility of the practitioner. Over 79% (626) felt that their organisation should have input into what comprised their CPC, a view that was supported somewhat by 43% (344) disagreeing with the regulatory body alone controlling composition of CPC (although 21% (166) favoured this).

### Consultation regarding specific models of continuous professional competence

Overall, the majority of respondents (77%) favoured the introduction of CPC by the regulatory body using a ‘mixed’ model approach of combining ‘mandatory’ and ‘voluntary’ activities, with 77% supportive of minimum standard requirements that include evidence of Patient Care Report (PCR) completion, clinical practice guidelines (CPGs) compliance and patient management.

Most respondents considered practical-type learning relevant to their roles (Table 2): practical training scenarios 94% (582), annual cardiac re-certification 92% (566), access to e-learning followed by related practice 90% (566), and training on simulation manikins 88% (535). The activities that received the highest ‘not relevant’ response were: ‘e-learning modules only and no related practice 36% (210); project work 27% (166); appraisal of journal publications 24% (147).

**Table 2:** Relevance of potential CPC activities

**Very relevant/Relevant = Relevant**	***Relevant***		***Not relevant***		**Total responses for question**
**Not relevant/Very irrelevant = Not relevant**					
	**Responses**	**% of total responses**	**Responses**	**% of total responses**	
Practical training scenarios	582	94%	9	1%	613
Annual cardiac re-certification	566	92%	29	4%	616
Access to e-learning followed by related practice	555	90%	11	2%	617
Access to medical journals/Medical books	538	88%	17	3%	615
Training on a simulation manikin	535	88%	25	4%	613
Attending courses accredited by PHECC	518	84%	30	5%	614
Annual cardiac first response revalidation	517	85%	60	10%	611
Evidence of current CPG compliance	489	80%	25	4%	611
Mentoring others	483	79%	47	8%	613
Major incident/Emergency exercises	480	78%	34	5%	612
Regular practical assessments	458	75%	48	8%	613
Working in a related hospital department	453	74%	64	10%	612
Keeping a portfolio of CPC activities	441	73%	55	9%	606
Relevant conferences e.g RESUS	405	66%	74	12%	613
Lecturing/Teaching	403	65%	76	12%	612
Appraisal with senior training officer (or above)	373	61%	92	15%	612
Being a tutor	349	57%	95	16%	607
Appraisal with a doctor/Medical supervisor	309	51%	115	19%	610
Being an examiner	309	51%	116	19%	607
Case study review	283	46%	114	19%	610
Project work	223	37%	166	27%	607
e-learning modules only and no related practice	203	33%	210	36%	607
Appraisal of journal publications	188	31%	147	24%	607

In addition to the practical-type, hands-on activities preferred for CPC maintenance, paramedics and APs also considered the following activities very relevant or relevant in maintaining Continuous Professional Competence: access to medical journals/books 88% (538/615); attending courses accredited by the Regulator (PHECC) 84% (518/614); annual Cardiac First Response (CFR) revalidation 85% (517/611); evidence of current CPG compliance 80% (489/611); mentoring others 79% (483/613); major incident/emergency exercises 78% (480/612); regular practical assessments 75% (458/613); working in a related hospital department 74% (453/612); keeping a portfolio of CPC activities 73% (441/606); attending relevant conferences 66% (405/613); lecturing/teaching 65% (403/612); appraisal with a senior Training Officer 61% (373/612); being a tutor 57% (349/607); appraisal with a doctor/medical supervisor 51% (309/610); being an examiner 51% (309/607); case study review 46% (283/610).

## Discussion

While some literature reports the development of ambulance CPD programmes internationally [7,8] and while CPD is more likely to lead to a change in practice when a needs assessment has been conducted [16], literature that reports consultation with practitioners prior to the introduction of such programmes is limited.

This first study of attitudes towards professional competence among paramedics and APs in Ireland suggests a genuine enthusiasm for the introduction of CPC, with 77% indicating that CPC was of personal importance to them, 74% indicating that evidence of CPC should be a condition for registration to practice and 53% already maintaining a CPC portfolio and participating in CPC activities, although not currently mandated to do so (Figure 3).

Funding and available time have both been identified in previous studies of healthcare workers as a barrier to CPD [17]. While 40% stated they had paid for CPC activities themselves, 65% would prefer if funding was provided and for them to arrange their own personal CPC activities, while 69% believed that the regulator should provide funding for organisations to develop their own customised CPC activities.

### Demographics

The majority of responses were from males and from registrants within the National Ambulance Service. This is unsurprising as the ambulance services in Ireland are provided, predominately, by the National Ambulance Service (NAS). In addition to the NAS, Dublin Fire Brigade provides ambulance services through twelve ambulances based throughout Dublin City. Both services are predominately male dominated.

### CPC activities

This survey identified a number of useful topics and activities that could be considered for the purpose of CPC and has identified some areas of low CPC priority for registrants.

Our survey included 23 potential CPC activities (Table 2) and asked which activities participants believed were relevant/irrelevant. Practical hands-on, training using simulation manikins, team-based activities or e-learning followed by practical skills were preferred over non-practical/theory-type activities. Also, there were less negative responses regarding activities related to practical skills than to theoretical skills. A study with Irish APs reinforced the concept of practical-type learning as a preferred methodology and as an effective way of maintaining competence [6], indeed scenario-based simulations have been used since 2007 as part of routine continuing education programmes by some American emergency medical services [9]. Interactive methods, for the purposes of CPD, such as team-based learning and case-based learning, as compared to lectures, impart sustainable knowledge and lead to high satisfaction among participants [18]. Davis *et al*. [19] in their systematic review found that interactive and mixed educational sessions were associated with a significant effect on physicians’ performance, effected change in professional practice and, on occasion, healthcare outcomes.

The least relevant activities were associated with non-skills/practical, individually-based, passive activities: e-learning modules only and no related practice 36% (210), project work 27% (166), appraisal of journal publications 24% (147). There is an interesting anomaly between appraising journal publications (24%) and having access to medical journals (88%). It is possible that some respondents may not critically assess the quality of journals but may still recognise the benefit of being able to access publications when seeking specific information. This is quite different to results seen from other professions who have tended to prefer attending conferences, lectures and reading of relevant journals [14,20] even though there is little evidence to suggest that attending conferences had any direct impact on improving professional practice [21].

Studies on cardiac nurses and Dietitians [14,22] have shown that journal reading was a popular preference and yet, for doctors, the effectiveness of continuous medical education (CME) increases as the intervention strategy becomes more active while activities classed as passive are not associated with changes in physician performance or patient outcome [21].

### Model of CPC

Groups were split in relation to opinion on annual hours of CPC; 35% (194) believed that 21-40 hours, while 26% (146) believed 41-60 hours were adequate. 77% of the respondents (474) favoured a ‘mixed’ model approach for CPC with a similar amount supporting the idea of minimum standard requirements which involved evidence of patient care. This ‘mixed’ model approach would allow for a ‘compulsory’ element to the CPC requirements and an additional ‘voluntary’ allowance that is still required but would allow the registrant some flexibility in deciding which activities to choose.

The benefit of mandatory CPD in healthcare professions has been debated. O’Connor’s [23] study on motivating factors for nurses participating in continuing education (CPD) suggested that the mandatory nature of the education had little influence in motivating participation, while Lee *et al*. [24] found that 66% of Australian radiographers thought CPD should be voluntary. Tellingly, Friedman and Woodhead [25] suggested that those professional bodies utilising compulsory or mixed policies with respect to CPD were likely to be promoting CPD as a means of maintaining competence.

Regarding sanctions, 61% (487) agreed that the practitioner should not be allowed to re-register at that level while 39% (307) agreed that those practitioners who fail to meet the PHECC CPC requirements should be allowed to register at the level below their current registration level. This finding is higher than from some other healthcare professions: 42% of pharmacists surveyed [26] favoured sanctions yet few dietitians favoured disciplinary action for those who failed to meet the registration requirements [22].

### Methodological considerations

While this first study of attitudes towards CPC among paramedics and APs in Ireland involved a national sample, we acknowledge some methodological considerations may limit generalisability. For instance, while we report data from 789 responses, this represented 43% of all registered paramedics and APs. Our study was limited to those with valid email addresses and clearly those for whom the subject area was a priority. Further research following the introduction of CPC for Irish paramedics and APs may expand upon these findings.

## Conclusions

There is a paucity of research conducted with registered pre-hospital practitioners in Ireland. This survey is the first to ascertain the opinions of paramedics and APs regarding CPC. This study further suggests that there is willingness on behalf of Irish paramedics and APs to engage with CPC, which is viewed as extremely important. Respondents considered it appropriate to link CPC with registration to practice and that there should be sanctions against those who do not meet CPC requirements.

The results of this survey demonstrate, at the very least, that emphasis will need to be placed on a compulsory ‘mixed’ model approach of CPC which includes evidence of patient contact and CPC activities that are practically orientated: practical training scenarios; annual cardiac recertification; e-learning followed by related practice; training on simulation manikins. Conversely, there is less interest in non-skills/practical, individual passive learning activities: e-learning alone and no related practice; project work, journal reviews. Somewhere between twenty to sixty hours of CPC activities per annum would appear to be acceptable to Irish practitioners as groups were split in their opinion: 35% (194) believed that 21-40 hours were adequate, while 26% (146) believed 41-60 hours were adequate. Arguably, the Regulatory Body (PHECC) might initially target the lower requirement with an expectation of 21-40 hours which is in-line with the small majority, and subsequently modify this requirement upwards following a review of the first cycle of CPC.

## Competing interests

The authors declare that they have no competing interests.

## Authors’ contributions

SK conceived of the study and was involved in the design, collection of data, data analysis, drafting the manuscript. WC and CD (principal investigator) were involved in the conception of the study, data analysis and interpretation and drafting the manuscript. All authors read, reviewed the manuscript critically for intellectual content, and approved the final manuscript.

## Pre-publication history

The pre-publication history for this paper can be accessed here:

http://www.biomedcentral.com/1472-6920/14/41/prepub
